# Influence of Gelatin and Propolis Extract on Honey Gummy Jelly Properties: Optimization Using D-Optimal Mixture Design

**DOI:** 10.3390/gels10040282

**Published:** 2024-04-21

**Authors:** Kultida Kaewpetch, Saowapa Yolsuriyan, Terd Disayathanoowat, Patcharin Phokasem, Taruedee Jannu, Gerry Renaldi, Rajnibhas Sukeaw Samakradhamrongthai

**Affiliations:** 1 Food Science and Technology Program, Faculty of Agro-Industry, Prince of Songkla University, Hat Yai, Songkhla 90110, Thailand; kultida7623@gmail.com (K.K.); saowapaying171041@gmail.com (S.Y.); taruedee404@gmail.com (T.J.); gerryren77@gmail.com (G.R.); 2 Research Center of Deep Technology in Beekeeping and Bee Products for Sustainable Development Goals (SMART BEE SDGs), Chiang Mai University, Chiang Mai 50200, Thailand; terd.dis@gmail.com (T.D.); patcharin.phokasem@gmail.com (P.P.); 3 Division of Product Development Technology, Faculty of Agro-Industry, Chiang Mai University, Chiang Mai 50100, Thailand

**Keywords:** *Apis cerana*, gummy jelly, honey, propolis

## Abstract

Gelatin is commonly used as a gelling agent in gummy candy. Honey and bee products are valuable and rich sources of biologically active substances. In this study, the influence of gelatin and propolis extract on honey gummy jelly (HGJ) properties was investigated. Honey (28–32%), xylitol (13–17%), and gelatin (6–10%) were utilized to develop HGJ products by mixture design methodology. Subsequently, the optimized formulation of HGJ was fortified with 1% and 2% propolis extract to enhance its phytochemicals and antimicrobial activities. The variation in the ingredients significantly affected the physicochemical, textural, and sensory properties of the HGJ. The optimized HGJ formulation consisted of honey (32%), xylitol (14%), and gelatin (7%) and exhibited 13.35 × 10^3^ g.force of hardness, −0.56 × 10^3^ g.sec of adhesiveness, 11.96 × 10^3^ N.mm of gumminess, 0.58 of resilience, and a moderate acceptance score (6.7–7.5). The fortification of HGJ with propolis extract significantly increased its phytochemical properties. Furthermore, the incorporation of propolis extract (2%) into the HGJ was able to significantly inhibit the growth of Gram-positive (*Streptococcus mutans* and *Staphylococcus aureus*) and Gram-negative (*Escherichia coli*) bacteria. The mixture of gelatin, xylitol, honey, and propolis extract can be utilized to develop a healthy gummy product with acceptable physicochemical, textural, and sensory qualities.

## 1. Introduction

Gummy jelly is a type of sugar confectionary that is based on a hydrocolloid that creates a network to hold sugar syrup with a comparatively high moisture content. The ingredients of gummy jelly typically include hydrocolloids as a gelling agent (e.g., gelatin and pectin), sweeteners (e.g., sucrose and glucose syrup), acids, flavorings, and coloring agents [1,2]. Gummy products are quite popular among younger consumers because of their organic and chewy qualities. However, because jellies and gummies include a lot of sugar and food additives, excessive and widespread use of them is regarded to be harmful to public health [3]. High rates of obesity, dental decay, and hyperglycemia have been linked to these items [4]. In response to customer demands for better formulations, companies are under increasing pressure to cut the sugar in these goods, as their low nutritional qualities have also been questioned [3]. Global confectionery trends indicate that low-calorie, sugar-free, and/or phytochemical-enriched confections are becoming more popular, as demonstrated by Konar et al. [5]. In producing healthier gummy jelly products, conventional sweeteners can be substituted with other sweetening products, such as honey [6].

Honey is a widely used bee product, which contains 80–95% sugar, as a sugar substitute in the food and beverages industry [7]. The bioactive compounds of honey are amino acids, vitamins, phenols, flavonoids, fatty acids, and organic acids. Furthermore, honey’s bioactivity in diabetes, cardiovascular disorders, cancer, hypertension, allergies, antioxidants, and prebiotic properties have been identified [6,7]. Propolis, more than any other bee product, needs specific attention. It is a sticky, viscous material that resembles resin that bees make from a combination of plant resin and insect secretions [8]. Customers are becoming more and more appreciative of bee products, such as propolis, because of their high concentration of bioactive chemicals, of which polyphenolic compounds—which are naturally occurring antioxidants—are a significant subset [7,8]. In this study, we used honey and propolis from *Apis cerana.* This Asian honeybee, *Apis cerana*, is the third smallest of the nine species of honeybees that are native to this area [9]. *Apis cerana* is a significant component of bee-keeping operations nowadays, particularly in honey production in Thailand.

Honey is not the only substitute sweetener utilized in creating low-calorie products. Sugar alcohols are carbohydrates that have a sweet taste and some sweet characteristics that remain between those of sugar and alcohol. They are also referred to as “polyols” or sugar replacements. The FDA views them as generally recognized as safe (GRAS) or as permitted food additives. To get the desired sweetness and flavor, these reduced-calorie sweeteners are commonly used with other sweeteners. Xylitol is one type of polyol that is widely used to replace sugar in culinary goods. The American Dietetic Association recommends that a daily xylitol intake should not exceed 0.37 g/kg for men and 0.42 g/kg body weight for women [10].

Much research has reported that the functionality of gummy jelly products can be increased by adding functional ingredients. Renaldi et al. [1] utilized *Garcinia atroviridis* puree into pectin/gelatin gummy jelly product. Teixeira-Lemos et al. [3] develop healthy gummy jellies containing natural fruits. Bee products were also found to hold the possibility of being added to gummy jelly products, as found by Rivero et al. [6]. However, the utilization of different sweeteners and functional ingredients affected the rheological and mechanical properties of gelatin gel [11,12]. In addition, the texture properties and mechanical behavior of gelatin gummy are affected by the concentration of gelatin [6,13,14]. Rivero et al. [6] found that utilizing more than 10% of gelatin as the gelling agent of gummy jelly product resulted in a firmer gummy product compared to gummy jelly product utilizing a lesser amount of gelatin. Therefore, the purpose of this study was to investigate the influence of gelatin and propolis extract on honey gummy jelly (HGJ). The physicochemical, textural, and sensory attributes of the formulated HGJ were tested. This study also assessed the fortification of propolis extract in the final formulation of HGJ in terms of its antimicrobial activity.

## 2. Results and Discussion

### 2.1. Just-About-Right (JAR) Analysis on Basis HGJ Formulation

**Figure 1:**
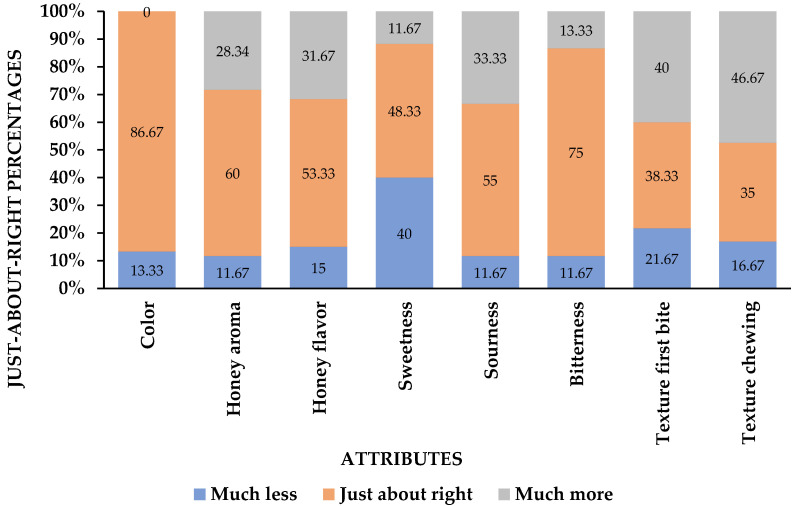
Just-About-Right (JAR) scale percentage of the basis HGJ formulation.

Prior to the development of HGJ, a basic formulation of honey gelatin gummy was made using Renaldi et al.’s [1] approach. The basis HGJ formulation consisted of honey (30%), xylitol (17%), gelatin (6%), citric acid (0.3%), and water (46.7%). Figure 1 shows the percentage of panelists giving consumer ratings on the attributes of the HGJ formula based on a collapsed JAR scale regarding the color, honey aroma, honey flavor, sweetness, sourness, bitterness, texture (first bite), and texture (chewing).

As shown in Figure 1, the JAR results on the color and bitterness exceeded 70%, so these attributes were excluded from penalizations. Furthermore, the penalizations on the honey aroma, honey flavor, and texture first bite value were excluded due to the net effect value being lower than 20%. In those categories where the number of responses was below the threshold of 20%, the penalizations were not taken into account [15]. The remaining three attributes, sweetness (28.33%), sourness (21.67%), and texture’s “chewing” (30.00%), were subjected to penalization, and the range of ingredients in the formulation for further investigation was adjusted accordingly.

### 2.2. Optimization Results of HGJ

#### 2.2.1. Color Values of HGJ

To quantify color values, the sample’s lightness was denoted by L*, and its greenness/redness and blueness/yellowness were explained by a* and b*, respectively [1]. In this study, the L*, a*, and b* values of the HGJ were in the ranges of 29.72–49.97, 13.01–24.35, and 38.03–57.11, respectively (Table 1). The difference in the amount of honey, xylitol, and gelatin used in the formulation of HGJ significantly affected the color of the product. The greater L* value obtained was due to the inability of the polyols to participate in Maillard reactions due to their lack of a reactive aldehyde group [16]. Conversely, a lower L* value was obtained by the HGJ with a higher amount of honey and gelatin. Honey and gelatin participated in the browning reactions via Maillard reaction and caramelization, which happen when sugar and amino acids are heated to high temperatures together. The browning reaction also results in the creation of a reddish-brown color, as indicated by an increase in the a* value of the HGJ [2].

#### 2.2.2. Texture Profile Analysis (TPA) of HGJ

The TPA is a method frequently employed in the industry to assess food textural behavior since it can reveal sensory eating characteristics [17]. According to Renaldi et al. [1], Rivero et al. [6], and Gunes et al. [17], hardness is one texture characteristic of gelled confections that is defined as the strength of a material’s gel structure under compression. The hardness value of the HGJ in this study was in the range of 10.03–31.35 × 10^3^ g.force. Increasing gelatin concentration promotes a significantly higher hardness value. Higher gelatin amounts in the formulation of gummy jelly led to increasing hardness due to the higher intermolecular contacts in the system [12]. Similarly, the same trend was found in the studies conducted by Renaldi et al. [1] and Tireki et al. [2].

Stickiness, or adhesiveness, is a term used to describe the amount of effort needed to resist the forces of attraction between the surface of the food and the surface of the object it comes into contact with, such as the tongue, teeth, or palate [18]. The effort needed to separate the probe from the sample following the initial compression is indicated by the TPA test. The observed adhesiveness of the HGJ was in the range of −1.23–−0.50 × 10^3^ g.force. Higher use of gelatin in this study was shown to exhibit significantly higher hardness and adhesiveness. These values are dependent upon the molecular structure of the products [12,18], as well as the surface qualities and the combined influence of adhesive and cohesive forces [19]. Research has indicated that bean jellies with a high adhesiveness value also have a high hardness value [18].

Springiness, which is also referred to as elasticity, is the speed at which a deformed sample regains its original state following the removal of the deforming force. This characteristic is crucial for determining how rubbery a jelly is in the mouth during food sensory testing since it indicates how much the gel structure is broken down during the initial compression [19]. It was observed that the springiness of HGJ with different honey, xylitol, and gelatin was in the range of 0.77–1.00 (Table 1). Gelatin concentration and springiness are correlated [19]; a higher gelatin content makes a product springier. According to Khouryieh et al. [20], jelly candy has a springiness that ranges from 0.90 to 1.50, and the findings of this investigation fall into that range. The springiness and hardness of food are inversely correlated; as firmness rises, elasticity falls [21].

The strength of a product’s internal linkages determines its cohesiveness, which indicates how well it resists a second deformation in comparison to the first. When cohesiveness is low (about 0.25), the product may be more likely to become moistened on the surface during storage. Therefore, cohesiveness, a measure of the attractive forces between similar molecules and, hence, structural integrity, should be taken into consideration and measured fresh immediately after production [2]. The measured cohesiveness value of the HGJ in this study was in the range of 0.67–0.78, significantly affected by the varied ingredients. The high value of cohesiveness indicated the HGJ was less prone to be moistened on the surface during storage.

The calculation of gumminess, a secondary parameter, involved multiplying hardness by cohesiveness, while chewiness was determined by multiplying hardness, cohesiveness, and springiness [1]. These two parameters are common texture descriptors for gelled confections, along with hardness [17]. The gumminess and chewiness exhibited by the HGJ were in the range of 7.29–24.34 × 10^3^ N.mm and 5.76–23.03 × 10^3^ N.mm, respectively. Research indicates that products become gummier as they get harder [18]. This is because harder jelly requires more energy to break down into a state that can be swallowed [21]. In addition, chewiness is a crucial textural attribute for a jelly product since it symbolizes the effort needed to masticate a solid item into a form that is ready to be swallowed [22]. As with gumminess, chewiness rises with increasing hardness [17].

The ability of the sample to regain its original shape and velocity after deformation is measured. It is, to put it simply, the elastic recovery of the sample. It was found that HGJ exhibited a resilience value in the range of 0.44–0.66 and was significantly affected by the varied ingredients. Elastic recovery capacity is correlated with the type of network that forms during the gelatin gel process, which is correlated with the molecular weight distribution, imino acid content, and extraction technique used [23]. The lower gelatin amount used in the formulation resulted in a lower imino acid content in the system, providing the product with lower resilience.

In terms of the sweeteners used in this study, it was found that honey and xylitol affected the texture of the HGJ. Cosolutes, such as sugars, including polyols and/or other carbohydrates, can have a significant impact on the behavior of the gelatin gels. According to Cebin et al. [24], the sugar profile and concentration are crucial for the gelling qualities of these systems. Wang and Hartel [25] have elucidated that the mechanical attributes, or texture, of hydrocolloid-prepared confections are contingent upon the interplay between the gelling agents, water, and sugars, in addition to the creation of junctions during the gelling process. The following mechanisms underpin gelation in sugar-containing gelatin gels: (i) alteration of the hydrogen-bonding water structure; (ii) decrease in the number of available water molecules as a result of sugar hydration; and (iii) exclusion of sugar molecules from the outermost layer of polymeric hydrocolloid molecules, which results in their aggregation [26]. Similarly, other researchers found that substituting sucrose with alternative sweeteners in the formulation of confectionery influenced its texture. The lower amount of sucrose utilized in the gelatin-based gummy jelly formulation decreased the hardness value of the product [27]. Samakradhamrongthai and Jannu [28] also found that the increase in stevia and xylitol amounts promoted less three-dimensional structure due to a lesser total soluble solids of the product, resulting in the decreased texture values of velvet tamarind candy.

#### 2.2.3. Moisture Content and Water Activity of HGJ

The results of moisture content and water activity (aw) of HGJ are shown in Table 2. The moisture content of the HGJ was in the range of 27.82–30.96%, which was significantly affected by different amounts of honey, xylitol, and gelatin amount in the formulation. It was observed that the HGJ exhibited relatively high moisture content. The honey and xylitol affected the HGJ to show high moisture content altogether with the increase in water. This is due to the interaction of honey and sugar, along with proteins, enzymes, acids, and minerals that can hold and absorb the moisture within the molecules, while xylitol can enhance the absorption and retain moisture within the product [28]. Researchers stated that the moisture content of jelly candies was in the range of 18–22%, which indicated that the HGJ exhibited higher moisture content [3,17,29]. Higher moisture content possessed by a product has the possibility to impact its texture and enhance the abilities of microbes to grow within the product, affecting its shelf life [29].

According to Ergun et al. [29], dissolved sugars, added sweeteners (polyols), salts (caramel), and humectants in confections are the main factors that affect water activity, which is a collative property based on the quantity and size of molecules in water. It was found that the water activity of the HGJs was in the range of 0.65–0.74, also significantly affected by the varied ingredients. The absorption of water by the product is impacted by the inclusion of humectants in the composition of gummy jelly [28]. Humectants have hydroxyl groups that have a propensity to establish hydrogen bonds with water molecules, as explained by Ergun et al. [29]. Samakradhamrongthai and Jannu [28] discovered that increased water activity of gummy jelly products suggested polyol-induced crystallization. Confectionery water activity rises as a result of the crystal lattice’s exclusionary formation process. Furthermore, sugar alcohol exhibits a high rate of crystallization to produce polyol crystals, which has an impact on the liquid phase’s lowered concentration of dissolved solids. The range of water activity of the HGJ found in this study was in accordance with the statement of another researcher [29]. The same researcher also stated that the product with the aforementioned range was prone to the growth of some molds and yeasts.

#### 2.2.4. Total Phenolic Content (TPC), Total Flavonoid Content (TFC), and Antioxidant Activities of HGJ

In this study, the TPC and TFC of the HGJ were in the range of 200.89–224.25 mg GAE/g and TFC 31.01–38.63 mg CE/g, respectively. The varied amounts of honey, xylitol, and gelatin significantly affected the TPC and TFC of the HGJ. The increment of honey amount increased the TPC and TFC of HGJ. According to various studies [30,31], honey and bee products are rich in polyphenolic chemicals that have potent anti-inflammatory, biocidal, and anticancer properties. The main factors contributing to honey’s health benefits are its strong antiradical activity and reductive characteristics, which aid in the deactivation of free radicals [30,31,32].

Antioxidants prevent oxidative stress, which is connected to conditions like cancer, neurological illnesses, and high blood pressure, by blocking or postponing unfavorable oxidation events [33]. Three distinct assays for measuring antioxidant activity—DPPH, ABTS, and FRAP—were used in this study to assess the antioxidant properties of HGJ. These assays were chosen based on their specific strength and limitations. The reaction mechanism of DPPH and ABTS was mixed mode (Hydrogen atom transfer (HAT) and single electron transfer (SET)), while the mechanism of FRAP was based on the SET mechanism. In terms of the polarity of antioxidants, DPPH is compatible with hydrophobic antioxidants, whereas FRAP is compatible with hydrophilic antioxidants, and the ABTS assay is compatible with both types of antioxidants [34]. The DPPH method is widely used to measure antioxidant activity. Based on the DPPH assay, the antioxidant activity of HGJ was in the range of 5.37–8.32 µg TE/g. ABTS and FRAP are two more commonly used methods to measure antioxidant activity. The ABTS test measures the ability of a substance to scavenge ABTS radical cations and convert them into an inert state [34]. The FRAP assay is another antioxidant test that evaluates the reducing power of a sample. The investigation of antioxidant activity using ABTS and FRAP assays revealed that the antioxidant activities of HGJ were in the range of 28.29–41.64 µg TE/g and 126.87–140.22 µg TE/g, respectively. The antioxidant activities of the HGJ are significantly affected by the varied ingredients.

#### 2.2.5. Sensory Evaluation of HGJ

The evaluation of the sensory characteristics of the HGJ (Figure S1) showed that sensory rating scores were in the range of neither like nor dislike to like moderately for the color (5.6–7.2) attribute (Table 3). Formula 1 exhibited the lowest color values (5.6 ± 1.8), suggesting that the interaction between varied ingredients resulted in a lower color score evaluated by the panelist. The use of color as a sensory attribute is crucial for influencing consumer purchasing decisions [16]. Concerning the results on the positive L* values, the lighter color exhibited by the HGJ holds the possibility of being more liked by the consumer, as shown by the HGJ with the highest L* value, which exhibited the highest color value rated by the panelist.

The investigation also showed that the HGJ exhibited sensory rating scores in the range of neither like nor dislike to like slightly for the honey aroma (5.6–6.4), honey flavor (5.1–6.5), sweetness (5.1–6.7), and sourness (5.0–6.5) of the HGJ. The presence of organic acids was created when 5-HMF (5-hydroxymethylfurfural) compounds broke down during the fermentation process of honey during storage after the harvesting process, and acid sugar (lactonic acid) gave honey its strong sour flavor [6,30,31]. However, the increment of gelatin in the formulation led to a significant decrease in the value of these sensory attributes. The trapping and binding of volatile chemicals inside the gummy jelly matrix was the reason for the less pronounced flavor that the gummy jelly created with a higher amount of gelatin displayed [1,6]. Chemical and physical binding both had an impact on flavor release, with gelatin more so than other gelling agents (such as pectin) and better-trapped volatiles [16]. The outcomes were in line with those of other researchers [6,12], who demonstrated that a gummy jelly with less flavor intensity was produced with a reduced gelatin content.

Based on these results, different honey, xylitol, and gelatin in the HGJ formulation resulted in products with a sensory rating score ranging from moderately dislike to like slightly for the texture’s “first bite” (3.3–6.7) and texture’s “chewing” (3.3–6.8). The high amount of honey and gelatin led to the lower texture score rated by the panelists. Together, the structural characteristics of honey will affect how gelatin forms a matrix [3,35]. Furthermore, the overall liking score was in the range of 4.1–6.8. The high percentage of honey and gelatin resulted in a lower overall liking score of the HGJ.

### 2.3. Model Fitting for the Optimization of HGJ Formula

#### 2.3.1. Optimization Model

**Figure 2:**
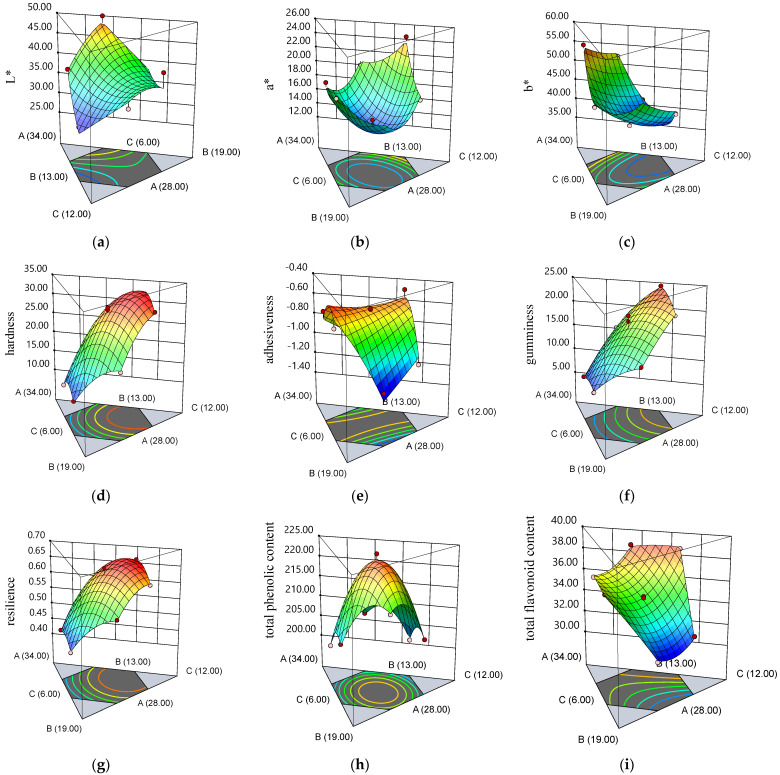
The response surfaces demonstrated that the correlation of honey (A), xylitol (B), and gelatin (C) corresponded to the following physical and chemical properties of HGJ: (**a**) L*; (**b**) a*; (**c**) b*; (**d**) hardness; (**e**) adhesiveness; (**f**) gumminess; (**g**) resilience; (**h**) total phenolic content; and (**i**) total flavonoid content.

**Figure 3:**
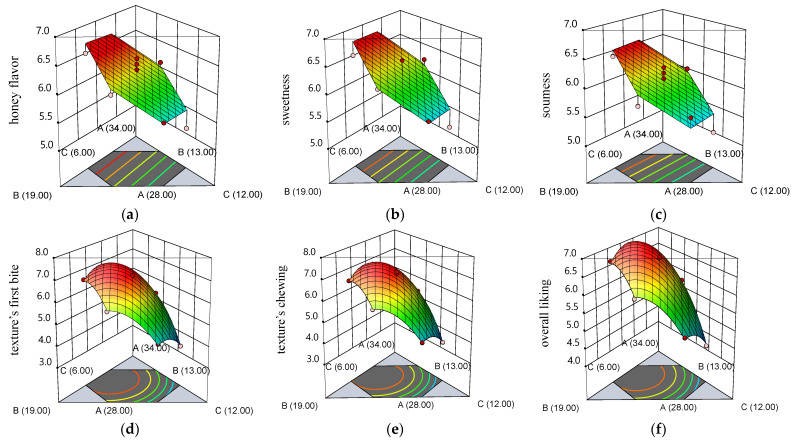
The response surfaces demonstrated that the correlation of honey (A), xylitol (B), and gelatin (C) corresponded to the following sensory attributes of HGJ: (**a**) honey flavor; (**b**) sweetness; (**c**) sourness; (**d**) texture’s first bite; (**e**) texture’s chewing; and (**f**) overall liking.

The physicochemical, textural, and sensory analysis was performed to optimize the honey, xylitol, and gelatin ratio in the formulation of HGJ. There were 15 responses from the optimization of the ingredients in the formulation of HGJ that could be generated using the regression equations (Table 4). The response surface of the significant HGJ properties generated the contour plots, as shown in Figure 2 and Figure 3.

The regression equation and contour plots for the significant physicochemical properties of the HGJ (Table 4 and Figure 2) revealed that honey affected the color properties of the HGJ positively, while its chemical and textural properties were affected negatively. The addition of xylitol showed a negative effect on the HGJ properties, except the a*, b*, and the TFC. Similarly, Renaldi et al. [1] and Cano-Lamadrid et al. [36] found that color played an important role in increasing the preference of consumers for jelly candies. In accordance with honey, the addition of gelatin increased the values of the properties of the HGJ, except adhesiveness, gumminess, chewiness, and TPC. According to Periche et al. [37], increasing the concentration of gelatin enhanced its toughness because more gelatin could stabilize the structure of the gel junction and hydrogen bond to hold the chain firmly. This resulted in a stable structure with density and a compact network. To create gels with both covalently cross-linked and microcrystalline regions, sugar sweetener/substitute, glucose syrup, and other trace elements can crosslink two gelatin molecules [11]. The R^2^ of the significant physicochemical and textural attributes HGJ were in the range of 0.7352–0.9874, with the *p*-value of the regression equations ranging from 0.0011 to 0.0480.

The regression equation and contour plots for the significant sensory properties of the HGJ are shown in Table 4 and Figure 3. The addition of honey and xylitol affected the sensory properties of the HGJ in the same way, positively affecting the honey flavor, sweetness, and sourness while negatively affecting the texture’s “first bite”, the texture’s “chewing”, and overall liking of the HGJ. In terms of gelatin, the increasing amount of gelatin decreases the values of all sensory properties of the HGJ. The R^2^ of the significant physicochemical and textural attributes HGJ were in the range of 0.7930–0.9740, with the *p*-value of the regression equations ranging from 0.0032 to 0.0089.

#### 2.3.2. Optimized HGJ Formulation

The optimized quantities of honey, xylitol, and gelatin were 32%, 14%, and 7%, respectively. The significant responses were validated with observation value using approximation error and exhibited approximation error in the range of 1.55–28.19%. The relatively high approximation errors were shown by the hardness (28.19%) and adhesiveness (26.02%), which contributed to the difference in the sample condition during TPA measurement prior to the optimization and during the validation process.

#### 2.3.3. Consumer Acceptance

A consumer acceptance test was performed on the HGJ made from the optimized formulation, using honey (32%), xylitol (14%), and gelatin (7%). The sensory rating scores from consumer acceptance (n = 400) showed that the optimized HGJ exhibited a moderate acceptance score in the range of like slightly to like moderately (6.7–7.5) (Figure S2), which indicated that the HGJ had desirable sensor attributes. Similar results were found on other sucrose-substituted gummy jelly tests performed by other researchers [6,14,36].

### 2.4. Fortification of Optimized HGJ with Propolis Extract

The fortification of extracted propolis powder to the HGJ showed a significant increase in the TPC, TFC, and antioxidant activities of the product, with no significant differences between extracted propolis concentrations (Table 5). According to the research by Habryka et al. [8] and Osés et al. [38], adding propolis to honey significantly increased the TPC and TFC. Propolis extracts were also a great source of antioxidants and other biologically active compounds. However, the enrichment of honey with propolis contributed to the deterioration of the sensory properties, as the changes in the sensory attributes of the HGJ were observed (Table 6). As seen in Table 6, the incorporation of 2% propolis extract into the HGJ formulation resulted in a sensory rating score in all attributes of the HGJ decreased, compared to the control and HGJ with 1% propolis extract. According to Habryka et al. [8], propolis should not be added to honey in amounts greater than 1% as it will negatively impact the sensory qualities of the honey.

**Figure 4:**
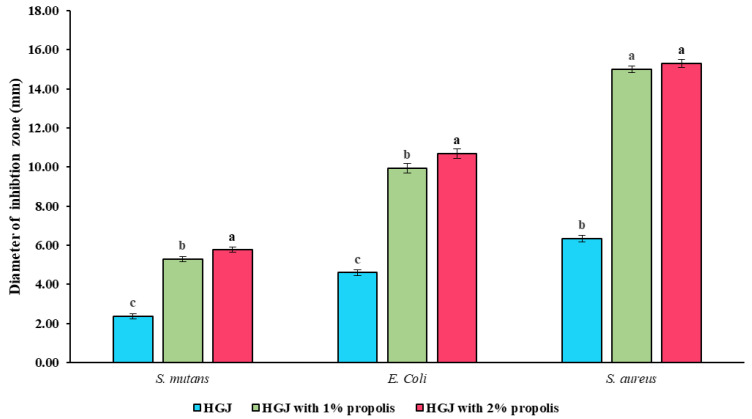
Antibacterial properties against *S. mutan* (DMST 1877), *S. aureus* (ATCC 25923), and *E. coli* (ATCC 8739) of HGJ and HGJ fortified with propolis extract (1% and 2% (*w*/*w*)) using agar well-diffusion method. Remark: the difference letter between sample means significant difference (*p* < 0.05).

Figure 4 shows the antibacterial properties of the propolis-fortified HGJ against a Gram-negative bacterium (*Escherichia coli*) and two Gram-positive bacteria (*Streptococcus mutans* and *Staphylococcus aureus*). Public health and food safety authorities are greatly concerned about food-borne pathogens and their toxins, and these three bacteria are thought to be the most important and commonly occurring worldwide [39]. Propolis has been shown to exhibit antibacterial activities against a broad range of pathogens, including viruses, bacteria, fungi, yeasts, and parasites [32,40]. When used in conjunction with honey, propolis has a synergistic impact that suppresses the growth of germs in microbiological cultures [30,32,40]. According to most research, propolis has substantial anti-Gram-positive action but only moderate anti-Gram-negative activity [39,41]. The varying cell wall structures of Gram-positive and Gram-negative bacteria could account for the differing capabilities of the propolis against them. Whereas Gram-positive bacteria lack an outside membrane and have a thicker peptidoglycan layer, Gram-negative bacteria have an outer membrane and a thinner layer [42]. As seen in the instance of *S. aureus*, propolis extract may disrupt the composition of cell walls or metabolic processes within cells, ultimately resulting in the death of Gram-positive bacteria. The comparatively increased resistance of the Gram-negative bacterium *E. coli* to the crude propolis extracts could be explained by the absence of an efficient target site in the metabolic or cellular structural components needed for destruction. Propolis extract has been shown to have greater antibacterial action against Gram-positive bacteria than against Gram-negative bacteria [42]. In terms of Gram-positive bacteria, the greater effect of the propolis-fortified HGJ on the *S. aureus* compared to the *S. mutans* was contributed by the shape of the bacterium. *S. mutans* are Gram-positive cocci that develop in pairs or chains, whereas *S. aureus* are Gram-positive cocci that prefer to form clusters on solid media [40].

## 3. Conclusions

This research revealed that honey can be utilized to develop a gummy jelly product with acceptable physicochemical and sensory characteristics. The use of honey, xylitol, and gelatin significantly affected the physicochemical, textural, and sensory properties of the HGJ. The optimized HGJ formula consisted of honey (32%), xylitol (14%), and gelatin (7%), providing a moderate acceptance score in the range from like slightly to like moderately (6.7–7.5). The fortification of the HGJ with extracted propolis powder further enhanced the phytochemical properties and antimicrobial activities of the product. The antimicrobial activity against two Gram-positive bacteria (*S. mutans* and *S. aureus*), and a Gram-negative bacterium (*E. coli*) was also found to be significantly enhanced by introducing propolis extract into the HGJ. The findings from this investigation are beneficial in developing a lower-sugar gummy jelly product with enhanced bioactivities and potential in the market, introduced as a healthy confectionery product beneficial for health. Nevertheless, further investigation on the utilized ingredients and processing methods could be performed to enhance the physicochemical properties and increase the sensory rating score of the product.

## 4. Materials and Methods

### 4.1. Materials

Honey and propolis were acquired from Beekeeping Farmer Community Enterprise (Chalung, Hat Yai, Songkhla, Thailand). The bacteria for the analysis of the microbial activity were obtained from the Thailand Institute of Scientific and Technological Research (TISTR). NaOH (PubChem CID 14798), ABTS (PubChem CID 16240279), DPPH (PubChem CID 57654141), and potassium persulfate (PubChem CID 24412) were obtained from Loba Chemie Pvt. Ltd., Mumbai, India. All analytical chemicals were attained from Avantor Performance Materials, LLC., Gliwice, Poland. All analytical grade solvents were procured from RCI Labscan Ltd., Bangkok, Thailand. All chemicals were of analytical grade.

### 4.2. Propolis Extraction

The propolis was extracted following the method by Nayaka et al. [9]. Raw Thai propolis materials were collected from *Apis cerana* Fabricius beehives (Songkhla, Thailand). The propolis was chopped into small pieces and extracted using 95% ethanol (1:5 *w*/*v*) at 25 °C, 300 rpm for 5 days. The mixture was filtered through Whatman™ no.1 filter paper, stored at −20 °C for 48 h, and filtered again. The filtrate was evaporated using a rotary evaporator (CH-9230, BÜCHI R200, Flawil, Switzerland) at 40 °C and stored at 4 °C, protected from light. The concentrated filtrate was dried using a freeze dryer (Freeze dryer system 7960032, Labconco, Kansas, KA, USA). The extracted propolis powder was stored in a vacuum seal package under 4 °C for further procedures.

### 4.3. Preparation of HGJ for JAR Analysis

In this study, the direction of the varied ingredients in the formulation of HGJ, honey, xylitol, and gelatin, was adjusted using the “Just-About-Right (JAR)” analysis, and the HGJ preparation method followed the method described by Renaldi et al. [1], with some modification. The basis mixture consisted of honey (30%), xylitol (17%), gelatin (6%), citric acid (0.3%), and water (46.7%) and was heated at 70 °C for 4 min. The mixture was heated until the brix reached approximately 70–75°Brix. After reaching the designated brix, the mixture was poured into a silicone mold and stored at 4 °C for 24 h before the JAR analysis.

### 4.4. Optimization of HGJ Formulation

After the basic formulation of the HGJ was found by using the JAR analysis, the honey (28–32%), xylitol (13–17%), and gelatin (6–10%), with the total percentage was 53%, were subjected to generate HGJ formula using Mixture Design (D-optimal), as shown in Supplementary Table S1. The gummy preparation was performed following Section 4.3. The HGJ was subjected to physicochemical, textural, and sensory evaluation for the optimization of HGJ.

### 4.5. Propolis Fortification to the Optimized HGJ Formulation

The propolis was fortified into the HGJ by adding the propolis extract (1% and 2%) with other ingredients during the processing of HGJ. The propolis-fortified HGJ was subjected to the analysis of its TPC, TFC, antioxidant activities, sensory evaluation, and antimicrobial properties.

### 4.6. Analysis Methods

#### 4.6.1. Color Measurement

The color values were measured following the method described by Renaldi et al. [1]. The color values L* (lightness), a* (greenness-redness), and b* (blueness-yellowness) of the HGJ were measured using a colorimeter (Color Quest XE, Color Global, Reston, VA, USA). The measurements were performed in triplicate.

#### 4.6.2. Texture Profile Analysis (TPA)

The TPA of the HGJ (hardness, cohesiveness, adhesiveness, springiness, gumminess, chewiness, and resilience) was conducted using the method described by Renaldi et al. [1] with 10 replications. The texture of the HGJ was analyzed using a texture analyzer model TA–XT plus (Stable Micro System, Surrey, UK) equipped with an SMS5 cylinder probe (35 mm). The analysis was performed at room temperature, and texture analysis device parameters were a pre-test speed of 1 mm/s, test speed of 5 mm/s, post-test speed of 5 mm/s, a strain of 75%, trigger force of 5 g, and the delay between two compressions was 3 s.

#### 4.6.3. Moisture Content and Water Activity

The moisture content of the HGJ was determined using the AOAC method 930.15, and the water activity was measured using a hygrometer (Aqualab, Decagon 3 TE, Pullman, WA, USA) at 25 °C [1]. The moisture content and water activity measurements were performed in triplicate.

#### 4.6.4. Total Phenolic Content (TPC), Total Flavonoid Content (TFC), and Antioxidant Activities

The preparation of the HGJ extract (with and without propolis extract) for the analysis of TPC, TFC, and antioxidant activities was performed according to the method by Samakradhamrongthai et al. [16]. The HGJ (5 g) was extracted using 50 mL of ethanol 60% (*v*/*v*) and stirred for 30 min at room temperature. The liquid was separated, and the extraction was repeated four more times. The liquid parts from all extractions were mixed and filtered to separate the wax. The extract was stored in an amber bottle at 4 °C until further analysis. The measurement was performed in triplicate.

1. TPC

The TPC of the HGJ was determined using the Folin–Ciocalteau method [16]. The methanolic extract (400 μL) was mixed with Folin–Ciocalteau phenol reagent (2 mL) and 7% Na_2_CO_3_ (1.6 mL). Gallic acid with different concentrations (0–150 mg/g) was used to create the standard curve. The mixed solution was allowed to stand for 45 min in the dark. The absorbance of the solution was measured at 765 nm, and the result was reported as mg of gallic acid equivalent (GAE)/g;

2. TFC

The TFC of the HGJ was estimated by mixing the extract (1 mL) with 5% NaNO_2_ solution (300 μL). After 5 min of being incubated at room temperature, 5% AlCl_3_ solution (300 μL), followed by 1 M NaOH solution (2 mL), was added to the mixture. Immediately, distilled water was added to the mixture until the volume was 10 mL, thoroughly mixed, and the absorbance was determined at 510 nm [16]. Catechin with different concentrations (0–50 mg/g) was used to create the standard curve, and the result was expressed as mg catechin equivalents (CE)/g;

3. Antioxidant activities

In this study, 3 different antioxidant activity assays, DPPH, ABTS, and FRAP, were employed to measure the antioxidant activities of HGJ. The absorbance value was measured using a spectrophotometer (Biochrom Libra S22, Biochrom, Cambridge, UK). The results on all assays were reported as mg trolox equivalent (TE)/g.

A solution consisting of DPPH (2.4 mg) in methanol (100 mL) was prepared for the DPPH radical scavenging assay. The DPPH radical scavenging activity was measured by mixing the extract (150 μL) with the methanolic DPPH (3.85 mL) and allowing for it to stand in the dark for 30 min. Trolox with different concentrations (0–60 mg/g) was used to create the standard curve. The absorbance was immediately measured at 517 nm [16].

An ABTS assay was conducted using a spectrophotometer (Biochrom Libra S22, Biochrom, UK). A working solution was obtained by mixing ABTS stock solution (7 mM ABTS in water and 2.45 mM potassium persulfate, 1:1). The solution was kept for 12–16 h at room temperature in the dark. The HGJ extract (150 μL) was mixed with the ABTS^·+^ solution (3.85 mL) and measured at 734 nm [16]. Trolox with different concentrations (0–500 mg/g) was used to create the standard curve.

The ferric reducing antioxidant power (FRAP) reagent for the FRAP assay was prepared by mixing 300 mM acetate buffer, 10 mM TPTZ in 40 mM HCl, and 20 mM FeCl_3_⋅6H_2_O (10:1:1) at 37 °C. The extract (150 μL) was mixed with the FRAP reagent (3.85 mL). The absorbance value was measured at 593 nm after 37 °C incubation using a water bath (Memmert, Schwabach, Germany) for 30 min [16]. Trolox with different concentrations (0–600 mg/g) was used to create the standard curve.

#### 4.6.5. Antimicrobial Activity of HGJ

The antimicrobial activity against *Staphylococcus aureus*, *Streptococcus mutan*, and *Escherichia coli* was tested via an agar well diffusion assay [43]. For this purpose, a 0.5 McFarland unit density suspension (~10^8^ CFU/mL) of each pathogenic bacterial strain was inoculated onto the surface of cooled Mueller–Hinton (MH) agar (Oxoid, Basingstoke, UK) using sterile cotton swabs. Afterward, five wells of 6 mm in diameter were punched in the agar. The ethanolic propolis-fortified HGJ extracts obtained from the phytochemical analysis preparation were filled in MH agar. Subsequently, pre-incubation was performed for 20 min at room temperature (25 °C) to allow for the diffusion of the samples into the media, followed by incubation at 37 °C for 24 h. The assays were run in triplicate, and samples that generated an inhibition zone (IZ) mean bigger than 10 mm were considered active against those specific bacteria. The diameter of the zone of inhibition in millimeters was measured.

### 4.7. Sensory Evaluation and Consumer Acceptance

The suitability of eight characteristics (honey aroma, honey flavor, color, sourness, bitterness, texture’s “first bite,” and texture’s “chewing”) of the HGJ was subjected to “just-about-right” (JAR) bipolar scales with five points (ranging from 5 = “much more” to 1 = “much less”) and a middle value of 3 = “just about right”. Subsequently, a 9-point hedonic scale [44] was used for the sensory evaluation and consumer acceptance, adding the overall liking of the HGJ to the original eight qualities. To do penalty analysis (PA), the original five-point scale was converted to a three-point scale. According to Ares and Varela [45] and Ortega-Heras et al. [15], the responses “more” and “much more” were grouped in a unique group titled “much more,” whereas the responses “much less” and “less” were grouped into a unique group named “much less”. The net effect value was obtained by calculating the differences between the “much more” and “much less” groups, with attributes possessing a net effect value >20% subjected to penalization.

The consumers were recruited from Hat Yai Municipality in Songkhla, Thailand, for the JAR (n = 50), the sensory evaluation for the optimization (n = 50), and consumer acceptance (n = 400) of the HGJ fortified with propolis extract. All participants had to sign a permission form and give written consent before this study. The samples underwent a monadic order of evaluation after being coded with a three-digit number [33]. The panelists were asked to stop and rinse their palates with room-temperature drinking water at the end of each sample. The ethical approval approved by The Prince of Songkla University Office of Human Research Ethics Committee authorized the same protocol, which was used for the sensory evaluations throughout this study (Approval No: HSc-HREC-63-045-1-1). This inquiry was conducted following the guidelines provided by the Helsinki Declaration.

### 4.8. Statistical Analysis

After analysis, each result was given as mean ± standard deviation. By highlighting the most negative attributes in terms of like, PA looked at the JAR data to identify potential areas for product enhancement based on consumer acceptability [30]. Using SPSS software version 17 (SPSS Inc., Chicago, IL, USA), the statistical analysis was carried out. The significance level at the 95% confidence limit was determined by applying Duncan’s Multiple Range Test (DMRT). The regression analysis was examined using Response Surface Methodology (RSM) (Design-Expert 13, Stat-Ease Inc., Minneapolis, MN, USA) to indicate the optimal amount of honey, xylitol, and gelatin in the formulation of HGJ.

## Figures and Tables

**Table 1:** Color value and textural properties of HGJ.

HGJ	Color Value			Hardness (× 10^3^ g.force)	Adhesiveness (× 10^3^ g.sec)	Springiness	Cohesiveness	Gumminess (× 10^3^ N.mm)	Chewiness (× 10^3^ N.mm)	Resilience
	L*	a*	b*							
1	29.72 ± 0.18 ^f^	24.35 ± 0.27 ^a^	38.33 ± 1.51 ^f^	31.35 ± 2.37 ^a^	−0.50 ± 0.31 ^a^	0.95 ± 0.04 ^ab^	0.78 ± 0.06 ^a^	24.34 ± 1.06 ^a^	23.03 ± 1.39 ^a^	0.66 ± 0.02 ^a^
2	49.97 ± 0.37 ^a^	19.01 ± 0.20 ^b^	57.11 ± 4.50 ^a^	10.03 ± 4.33 ^e^	−0.65 ± 0.31 ^a^	1.00 ± 0.55 ^a^	0.78 ± 0.16 ^ab^	7.29 ± 2.86 ^d^	6.44 ± 2.08 ^e^	0.46 ± 0.06 ^d^
3	35.27 ± 0.27 ^e^	13.01 ± 0.42 ^f^	38.03 ± 0.26 ^f^	27.46 ± 1.55 ^b^	−0.60 ± 0.07 ^a^	0.85 ± 0.04 ^ab^	0.70 ± 0.08 ^abc^	19.13 ± 1.31 ^b^	16.27 ± 1.47 ^bc^	0.63 ± 0.05 ^a^
4	36.36 ± 0.69 ^d^	18.94 ± 0.89 ^b^	45.58 ± 0.96 ^bcd^	10.33 ± 0.73 ^e^	−0.68 ± 0.23 ^a^	0.77 ± 0.04 ^b^	0.72 ± 0.04 ^abc^	7.46 ± 0.78 ^d^	5.76 ± 0.68 ^e^	0.44 ± 0.030 ^d^
5	39.51 ± 1.20 ^b^	20.00 ± 0.60 ^b^	52.00 ± 2.73 ^ab^	21.69 ± 4.32 ^c^	−1.05 ± 0.26 ^b^	0.79 ± 0.13 ^b^	0.72 ± 0.08 ^abc^	15.28 ± 2.52 ^c^	12.27 ± 3.13 ^d^	0.59 ± 0.04 ^b^
6	40.57 ± 0.49 ^b^	16.61 ± 0.71 ^d^	48.42 ± 4.58 ^bc^	18.55 ± 2.04 ^d^	−1.23 ± 0.15 ^b^	0.77 ± 0.05 ^b^	0.74 ± 0.04 ^abc^	13.67 ± 1.42 ^c^	10.59 ± 1.63 ^d^	0.55 ± 0.02 ^c^
7	36.79 ± 0.52 ^d^	14.46 ± 0.61 ^e^	40.74 ± 2.63 ^ef^	29.91 ± 1.54 ^ab^	−0.63 ± 0.09 ^a^	0.85 ± 0.03 ^ab^	0.68 ± 0.06 ^c^	20.23 ± 2.39 ^b^	17.17 ± 1.94 ^bc^	0.63 ± 0.04 ^a^
8	37.90 ± 0.67 ^c^	13.74 ± 0.92 ^ef^	40.13 ± 7.73 ^ef^	30.42 ± 3.78 ^a^	−0.59 ± 0.04 ^a^	0.77 ± 0.11 ^b^	0.67 ± 0.03 ^c^	20.41 ± 2.91 ^b^	15.68 ± 2.50 ^c^	0.66 ± 0.04 ^a^
9	35.00 ± 0.56 ^e^	17.76 ± 0.36 ^c^	42.09 ± 2.24 ^def^	30.18 ± 2.94 ^a^	−1.08 ± 0.34 ^b^	0.86 ± 0.03 ^ab^	0.70 ± 0.09 ^bc^	20.95 ± 2.47 ^b^	18.00 ± 2.14 ^b^	0.62 ± 0.02 ^ab^
*p*-value	<0.001	<0.001	<0.001	<0.001	<0.001	0.108	0.022	<0.001	<0.001	<0.001

Remarks: Data are shown as mean ± standard deviation. Different superscript letter means significant differences (*p* < 0.05) between treatments.

**Table 2:** Chemical properties of HGJ.

HGJ	Moisture Content (%)	Aw	TPC (mg GAE/g)	TFC (mg CE/g)	Antioxidant Activities		
					DPPH (μg TE/g)	FRAP (μg TE/g)	ABTS (μg TE/g)
1	28.84 ± 0.63 ^bc^	0.75 ± 0.022 ^a^	200.89 ± 1.31 ^c^	38.58 ± 0.60 ^a^	8.32 ± 0.11 ^a^	28.29 ± 0.33 ^d^	136.70 ± 2.28 ^abc^
2	28.22 ± 0.34 ^cd^	0.68 ± 0.008 ^c^	201.40 ± 2.64 ^c^	36.61 ± 0.25 ^b^	6.64 ± 0.12 ^c^	29.19 ± 0.52 ^d^	138.73 ± 1.83 ^ab^
3	30.96 ± 0.79 ^a^	0.74 ± 0.002 ^a^	224.25 ± 9.00 ^a^	35.25 ± 1.64 ^bc^	6.48 ± 0.21 ^cd^	41.42 ± 1.21 ^a^	135.15 ± 4.29 ^bcd^
4	29.57 ± 0.17 ^b^	0.67 ± 0.003 ^c^	205.97 ± 3.03 ^c^	36.46 ± 0.73 ^b^	6.90 ± 0.19 ^b^	29.76 ± 1.40 ^d^	140.22 ± 1.64 ^a^
5	27.82 ± 0.32 ^d^	0.67 ± 0.003 ^c^	206.65 ± 5.40 ^bc^	38.63 ± 1.10 ^a^	5.37 ± 0.06 ^e^	32.23 ± 2.00 ^c^	128.57 ± 1.21 ^fg^
6	28.86 ± 0.45 ^bc^	0.65 ± 0.004 ^d^	213.87 ± 4.49 ^b^	31.01 ± 1.51 ^d^	6.59 ± 0.14 ^cd^	35.79 ± 1.49 ^b^	132.40 ± 4.72 ^cdf^
7	27.86 ± 0.08 ^d^	0.67 ± 0.003 ^c^	221.58 ± 2.00 ^a^	34.34 ± 1.09 ^c^	6.39 ± 0.05 ^d^	41.54 ± 0.91 ^a^	126.87 ± 0.11 ^g^
8	28.08 ± 0.71 ^cd^	0.72 ± 0.001 ^b^	222.53 ± 1.23 ^a^	35.11 ± 1.06 ^bc^	6.39 ± 0.04 ^d^	41.64 ± 0.55 ^a^	127.24 ± 0.47 ^g^
9	29.50 ± 0.40 ^b^	0.66 ± 0.003 ^c^	205.14 ± 4.35 ^c^	32.05 ± 0.79 ^d^	8.27 ± 0.10 ^a^	29.90 ± 1.06 ^d^	131.26 ± 2.04 ^dfg^
*p*-value	<0.001	<0.001	<0.001	<0.001	<0.001	<0.001	<0.001

Remarks: Data are shown as mean ± standard deviation. Different superscript letter means significant differences (*p* < 0.05) between treatments.

**Table 3:** Sensory evaluation of HGJ.

HGJ	Color	Honey Aroma	Honey Flavor	Sweetness	Sourness	Texture’s First Bite	Texture’s Chewing	Overall Liking
1	5.6 ± 1.8 ^b^	5.6 ± 1.6 ^b^	5.1 ± 1.8 ^c^	5.1 ± 1.8 ^b^	5.0 ± 1.8 ^d^	3.3 ± 2.0 ^d^	3.3 ± 1.9 ^d^	4.1 ± 1.8 ^d^
2	7.0 ± 1.4 ^a^	6.1 ± 1.3 ^ab^	6.5 ± 1.0 ^a^	6.7 ± 1.1 ^a^	6.5 ± 1.2 ^a^	6.7 ± 1.2 ^a^	6.7 ± 1.2 ^a^	6.8 ± 0.9 ^a^
3	6.8 ± 1.5 ^a^	6.4 ± 1.2 ^a^	6.2 ± 1.6 ^a^	6.1 ± 1.5 ^a^	6.0 ± 1.3 ^abc^	6.3 ± 1.3 ^a^	6.3 ± 1.3 ^a^	6.3 ± 1.1 ^a^
4	7.1 ± 1.2 ^a^	6.2 ± 1.3 ^a^	6.5 ± 1.3 ^a^	6.5 ± 1.4 ^a^	6.4 ± 1.5 ^a^	6.5 ± 1.4 ^a^	6.4 ± 1.5 ^a^	6.6 ± 1.3 ^a^
5	7.1 ± 1.0 ^a^	5.9 ± 1.4 ^ab^	6.1 ± 1.5 ^a^	6.2 ± 1.4 ^a^	6.0 ± 1.7 ^abc^	5.3 ± 1.7 ^b^	5.3 ± 1.8 ^b^	5.7 ± 1.4 ^b^
6	6.7 ± 1.3 ^a^	6.0 ± 1.5 ^ab^	6.0 ± 1.6 ^a^	6.1 ± 1.5 ^a^	5.7 ± 1.5 ^ab^	5.6 ± 1.6 ^a^	5.6 ± 1.6 ^a^	5.9 ± 1.2 ^a^
7	7.1 ± 1.1 ^a^	6.1 ± 1.4 ^ab^	6.4 ± 1.6 ^a^	6.4 ± 1.6 ^a^	6.1 ± 1.5 ^ab^	6.7 ± 1.2 ^a^	6.7 ± 1.4 ^a^	6.7 ± 1.1 ^a^
8	7.2 ± 1.3 ^a^	5.9 ± 1.3 ^ab^	6.3 ± 1.3 ^ab^	6.4 ± 1.5 ^a^	6.2 ± 1.5 ^bc^	6.7 ± 1.3 ^b^	6.8 ± 1.1 ^b^	6.6 ± 1.1 ^b^
9	6.8 ± 1.1 ^a^	5.9 ± 1.6 ^ab^	5.5 ± 1.7 ^bc^	5.5 ± 1.6 ^b^	5.5 ± 1.5 ^cd^	4.1 ± 2.0 ^c^	4.0 ± 2.0 ^c^	4.8 ± 1.7 ^c^
*p*-value	<0.001	<0.001	<0.001	<0.001	<0.001	<0.001	<0.001	<0.001

Remarks: Data are shown as mean ± standard deviation. Different superscript letter means significant differences (*p* < 0.05) between treatments.

**Table 4:** Regression equation of significant responses using RSM.

Responses	Regression Equation	R^2^	*p*-Value
L*	+8.01 × A − 11.40 × B + 11.04 × C − 1.12 × A × C + 1.24 × B × C	0.7352	0.0480
a*	+7.57 × A + 30.11 × B + 45.63 × C − 1.14 × A × B − 1.16 × A × C − 1.98 × B × C	0.9259	0.0153
b*	+26.16 × A + 49.29 × B + 46.62 × C − 2.64 × A × B − 2.49 × A × C − 0.58 × B × C	0.9470	0.0094
Hardness (×10^3^ g.force)	−18.07 × A − 51.49 × B + 60.91 × C + 2.29 × A × B + 2.29 × A × C + 2.11 × B × C	0.9736	0.0033
Adhesiveness (×10^3^ g.sec)	−1.08 × A − 2.61 × B − 0.47 × C +0.13 × A × B +63.32 × A × C − 4.94 × B × C	0.8403	0.0471
Gumminess (×10^3^ N.mm)	−11.64 × A − 29.05 × B − 34.69 × C + 1.36 × A × B + 1.45 × A × C + 1.01 × B × C	0.9874	0.0011
Resilience	−0.14 × A − 0.39 × B − 0.72 × C + 0.02 × A × B + 0.03 × A × C + 0.02 × B × C	0.9673	0.0046
Total phenolic com-pound (mg GAE/g)	−91.34 × A − 270.19 × B − 620.04 × C + 12.53 × A × B + 19.52 × A × C + 19.36 × B × C	0.9844	0.0015
Total flavonoid com-pound (mg CE/g)	− 0.74 × A + 3.50 × B + 5.03 × C + 0.02 × A × B + 0.14 × A × C − 0.65 × B × C	0.9694	0.0041
Honey flavor	+0.15 × A + 0.17 × B − 0.14 × C	0.7930	0.0089
Sweetness	+0.16 × A + 0.16 × B − 0.16 × C	0.8169	0.0061
Sourness	+0.16 × A + 0.15 × B − 0.15 × C	0.8201	0.0058
Texture’s first bite	− 1.69 × A − 6.78 × B − 10.84 × C + 0.28 × A × B + 0.29 × A × C + 0.42 × B × C	0.9740	0.0032
Texture’s chewing	−1.76 × A − 6.99 × B − 11.57 × C + 0.29 × A × B + 0.31 × A × C + 0.44 × B × C	0.9707	0.0039
Overall liking	−1.04 × A − 4.74 × B − 7.47 × C + 0.19 × A × B + 0.19 × A × C + 0.30 × B × C	0.9550	0.0063

Remark: A = Honey, B = Xylitol, C = Gelatin.

**Table 5:** TPC, TF, and antioxidant activities of HGJ with propolis extract.

Sample	TPC (mg GAE/g)	TFC (mg CE/g)	Antioxidant Activities		
			DPPH (μg TE/g)	FRAP (μg TE/g)	ABTS (μg TE/g)
HGJ	224.25 ± 9.00 ^b^	35.21 ± 1.64 ^b^	648.47 ± 0.70 ^b^	41.42 ± 1.60 ^b^	135.15 ± 0.29 ^b^
HGJ with 1% propolis extract	323.70 ± 0.16 ^a^	45.40 ± 0.28 ^a^	704.20 ± 0.54 ^a^	44.80 ± 0.58 ^a^	148.81 ± 0.56 ^a^
HGJ with 2% propolis extract	313.93 ± 0.21 ^a^	45.53 ± 0.05 ^a^	705.31 ± 0.58 ^a^	45.46 ± 10.58 ^a^	149.15 ± 0.01 ^a^
*p*-value	<0.001	<0.001	0.002	0.001	0.002

Remarks: GAE = gallic acid equivalent; CE = Catechin equivalent; TE = Trolox equivalent. Different superscript letter means significant differences (*p* < 0.05) between treatments.

**Table 6:** Sensory evaluation of HGJ with propolis extract.

Sample	Attributes							
	Color	Honey Aroma	Honey Flavor	Sweetnessns	Sourness	Texture’s First Bite	Texture’s Chewing	Overall Liking
HGJ	7.8 ± 0.8 ^a^	7.4 ± 1.3 ^a^	7.4 ± 1.3 ^a^	7.3 ± 1.3	7.2 ± 1.2 ^a^	7.5 ± 1.3 ^a^	7.5 ± 1.3 ^a^	7.6 ± 1.2 ^a^
HGJ with 1% propolis extract	7.3 ± 1.0 ^b^	6.9 ± 1.3 ^b^	7.4 ± 1.1 ^a^	7.2 ± 1.1	7.1 ± 1.0 ^a^	7.1 ± 1.2 ^ab^	7.3 ± 1.2 ^a^	7.2 ± 1.2 ^a^
HGJ with 2% propolis extract	7.0 ± 1.1 ^b^	6.7 ± 1.0 ^b^	6.6 ± 1.3 ^b^	6.8 ± 1.2	6.5 ± 1.3 ^b^	6.6 ± 1.4 ^b^	6.6 ± 1.2 ^b^	6.6 ± 1.4 ^b^
*p*-value	0.002	0.003	0.001	0.100	0.011	0.005	0.002	<0.001

Remarks: Data are shown as mean ± standard deviation. Different superscript letter means significant differences (*p* < 0.05) between treatments. ns superscript letter means no significant difference (*p* > 0.05).

## Data Availability

The data generated during the current study are available from the corresponding author upon reasonable request.

## Supplementary Materials

The following supporting information can be downloaded at https://www.mdpi.com/article/10.3390/gels10040282/s1, Figure S1: The HGJ from 9 formulations; Figure S2: The consumer acceptance of HGJ with optimized formula; Table S1: Experimental design of HGJ.
