# Supplementary material for: Influence of Gelatin and Propolis Extract on Honey Gummy Jelly Properties: Optimization Using D-Optimal Mixture Design
Source: Gels. 2024 Apr 21;10(4):282. doi: 10.3390/gels10040282 (PMC11049484; doi:10.3390/gels10040282)
Supplement: Supplementary file 1 [file gels-10-00282-s001.zip › gels-2944478-supplementary.pdf]

Supplementary

Influence of Gelatin and Propolis Extract on Honey Gummy Jelly Properties: Optimization Using D-Optimal Mixture Design

Kultida Kaewpetch [1], Saowapa Yolsuriyan [1], Terd Disayathanoowat [2], Patcharin Phokasem [2], Taruedee Jannu [1], Gerry Renaldi [1,3] and Rajnibhas Sukeaw Samakradhamrongthai [2,3,*]

[1] Food Science and Technology Program, Faculty of Agro-Industry, Prince of Songkla University, Hat Yai, Songkhla 90110, Thailand; kultida7623@gmail.com (K.K.); saowapaying171041@gmail.com (S.Y.); taruedee404@gmail.com (T.J.); gerryren77@gmail.com (G.R.)
[2] Research Center of Deep Technology in Beekeeping and Bee Products for Sustainable Development Goals (SMART BEE SDGs), Chiang Mai University, Chiang Mai 50200, Thailand; terd.dis@gmail.com (T.D.); patcharin.phokasem@gmail.com (P.P.)
[3] Division of Product Development Technology, Faculty of Agro-Industry, Chiang Mai University, Chiang Mai 50100, Thailand
* Correspondence: rajnibhas.s@cmu.ac.th

Table S1. Experimental design of HGJ.

Treat-ment	Honey (A; %)	Xylitol (B; %)	Gelatin (C; %)
**1**	30	13	10
**2**	32	15	6
**3**	30	15	8
**4**	30	17	6
**5**	32	13	8
**6**	28	17	8
**7**	30	15	8
**8**	30	15	8
**9**	28	15	10

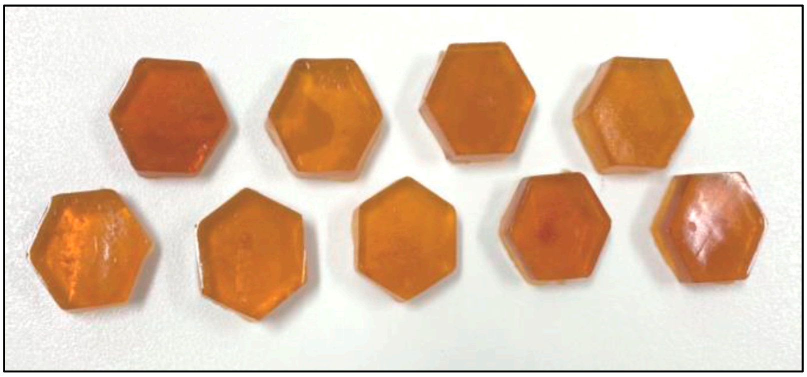

Figure S1. The HGJ from 9 formulations

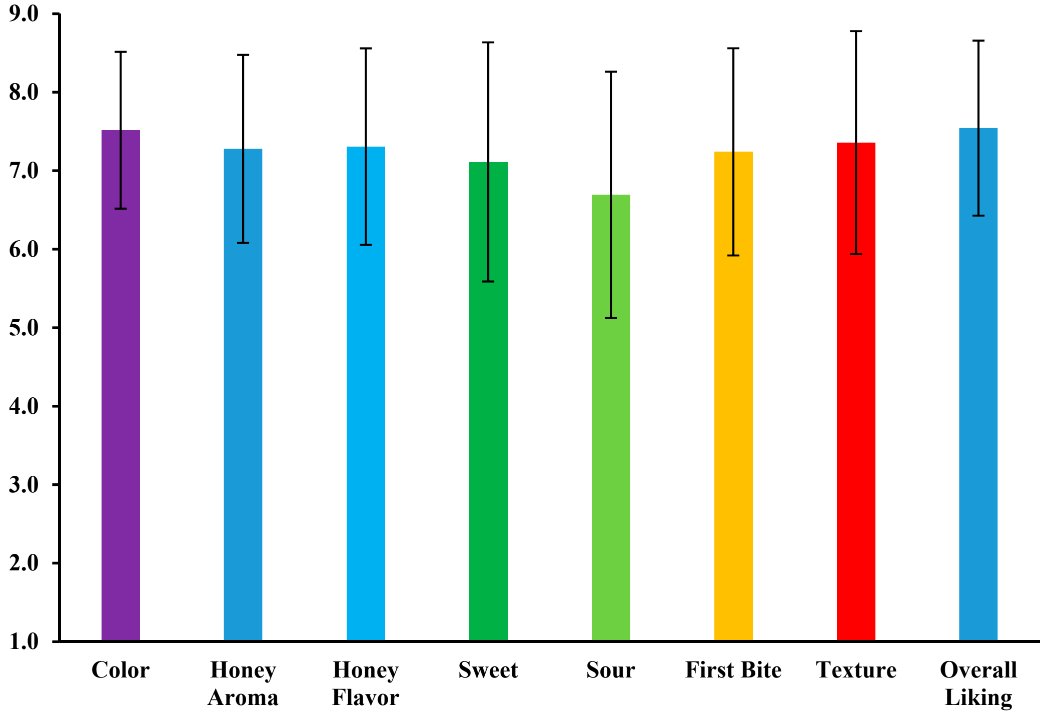

Figure S2. The consumer acceptance of HGJ with optimized formula
